# Supplementary material for: Estimation of Lifetime QALYs Based on Lifestyle Behaviors
Source: Int J Environ Res Public Health. 2021 Sep 22;18(19):9970. doi: 10.3390/ijerph18199970 (PMC8508078; doi:10.3390/ijerph18199970)
Supplement: Supplementary file 1 [file ijerph-18-09970-s001.zip › ijerph-1348265-supplementary.pdf]

Table S1. 95% CI for lifetime expected QALYs per capita in scenario analysis

QALYs per person		Physical Activity			
		Very often	Usually	Not so active	Too little
All					
Sleeping	Well	3.6-35.1	3.5-36.3	3.4-35.2	3.3-34.0
	Lack of sleep	3.4-34.9	3.3-33.8	3.2-32.8	3.1-31.6
Age 10–19					
Sleeping	Well	25.1-40.0	24.4-39.0	23.7-37.8	22.9-36.5
	Lack of sleep	2.4-37.4	22.8-36.3	22.1-35.2	21.2-33.9
Age 20–29					
Sleeping	Well	20.4-38.4	19.9-37.4	19.3-36.3	18.6-35.0
	Lack of sleep	19.1-35.9	18.5-34.8	18.0-33.7	17.3-32.5
Age 30–39					
Sleeping	Well	16.2-35.9	15.8-35.0	15.3-34.0	14.8-32.8
	Lack of sleep	15.2-33.6	14.7-32.6	14.3-31.6	13.8-30.5
Age 40–49					
Sleeping	Well	11.0-33.2	10.7-32.3	10.4-31.4	10.1-30.3
	Lack of sleep	10.3-31.1	10.0-30.2	9.7-29.2	9.3-28.1
Age 50–59					
Sleeping	Well	5.5-29.5	5.4-28.7	5.2-27.8	5.0-26.9
	Lack of sleep	5.2-27.5	5.0-26.7	4.9-25.9	4.7-25.0
Age 60–69					
Sleeping	Well	2.9-25.3	2.8-24.6	2.7-23.9	2.6-23.0
	Lack of sleep	2.7-23.6	2.6-22.9	2.5-22.2	2.4-21.4
Age 70–79					
Sleeping	Well	1.9-20.5	1.8-19.9	1.8-19.3	1.7-18.6
	Lack of sleep	1.7-19.1	1.7-18.5	1.6-17.9	1.6-17.3
